# Specificity of Saliva Esterases by Wine Carboxylic Esters and Inhibition by Wine Phenolic Compounds Under Simulated Oral Conditions

**DOI:** 10.3389/fnut.2021.761830

**Published:** 2021-11-04

**Authors:** María Pérez-Jiménez, Carolina Muñoz-González, María Ángeles Pozo-Bayón

**Affiliations:** Instituto de Investigación en Ciencias de la Alimentación (CIAL), Consejo Superior de Investiagciones Científicas-Universidad Autónoma de Madrid (CSIC-UAM), Madrid, Spain

**Keywords:** wine odorant esters, stimulated and non-stimulated saliva, oral aroma metabolism, saliva esterase enzymes, polyphenols

## Abstract

The specificity of human esterase activity (EA) from the stimulated (SS) and non-stimulated (NSS) saliva toward different typical wine odorant carboxylic esters and its inhibition by the wine phenolic compounds has been evaluated. For the specificity, six p-nitrophenyl linked esters with different carbon chain lengths (from 2 to 12 carbons) were employed. The five single phenolic compounds (catechin, quercetin, kaempferol, myricetin, and resveratrol) at typical wine concentrations were assayed in the salivary EA inhibition study. Additionally, the inhibition exerted by the mixtures of wine polyphenols was evaluated using four commercial phenolic extracts [a grape seed extract (GSE), the monomers and oligomer fraction of the GSE, and a red wine extract (RWE)]. Finally, the saliva EA under the wine consumption conditions (pH = 5 and 11.3% ethanol) was evaluated. The results showed a higher EA in SS than NSS. It was also shown that the EA was higher toward the smaller than bigger esters regardless of the saliva types (SS or NSS). However, the inhibition exerted on saliva EA by the individual and mixtures of phenolic compounds was proven. Catechin was the phenolic compound that mostly inhibited saliva EA, while resveratrol showed the lowest EA inhibition. This inhibition was mainly related to the concentration of the phenolic compounds, but also with its structure. Finally, under simulated wine consumption, a decrease in EA was produced, which was mainly provoked by the decrease in the salivary pH. Nonetheless, since salivary pH recovers a few seconds after wine consumption, saliva EA might be relevant for the long-lasting perception of wine esters.

## Highlights

- Esterase activity (EA) was higher in stimulated saliva than unstimulated saliva.
- Saliva EA was higher for the smaller carboxylic esters (C2, C4) than the bigger ones (>C6).
- The addition of wine polyphenols individually or as a mixture decreased saliva EA.
- The EA inhibition was related to the polyphenol concentration and structure.
- The saliva EA was compromised under the wine consumption conditions (pH 5, 11% ethanol).

## Introduction

Saliva plays a key role in the aroma perception of food and beverages since it can interact with the odorants during oral processing (1, 2). One of the mechanisms why saliva might impact aroma perception is through the metabolism of aroma compounds by the salivary enzymes (3–6). Among them, the saliva esterase enzymes are shown to hydrolyze esters into their corresponding carboxylic acids (7) and alcohols (4). This might impact the aroma perception since the new metabolites will have different odor thresholds and qualities to their corresponding esters. In the case of wine, the metabolism of esters in the oral cavity by saliva esterases could be of utmost importance, considering that this group of volatile odorant compounds are ubiquitous not only in wine, but in many fermented beverages, contributing to the fresh, fruity, and pleasant aromatic notes of these beverages (8).

The saliva esterases originate from different sources, such as salivary glands, oral microbiota, or epithelial cells (9). Because of this, depending on the method of saliva collection (stimulated or non-stimulated), the composition of saliva could vary, since stimulated saliva (SS) and non-stimulated saliva (NSS) are produced from different glands (10). In this regard, although numerous salivary parameters (proteins, antioxidant status, uric acid, etc.) are found at higher concentrations in the unstimulated saliva (11), the effect of the saliva collection methods is not investigated for saliva esterase activity (EA).

In addition, it is important to note that different types of enzymes could contribute to the total saliva EA, such as carboxylesterases (9), carbonic anhydrases (12, 13), cholesterol esterases (14), choline esterases (15), or lipases (16). Within them, the carboxylesterases have been reported to be the main contributors to the total EA in saliva (9, 17). However, some others works in which specific saliva esterase inhibitors were used, suggested that carbonic anhydrases IV was the main contributor to saliva EA (18).

In addition to saliva, the esterase enzymes are present in different human tissues and have wide substrate specificity (19, 20). Among others, they act on the carboxylic esters liberating their corresponding acids and alcohols. In the previous works, a preference of saliva esterase toward the longer than the shorter aromatic esters was proposed (3, 7), which seems to be contradictory to the fact that the longer esters are more persistent for a longer time in the mouth than the shorter esters after wine tasting (7, 21). Nonetheless, in the abovementioned works, the authors used an indirect measurement, determining the amount of aromatic esters released after their incubation with the saliva samples (3, 7). Similarly, Perez-Jiménez et al. (7) showed the presence of metabolic degradation products (hexanoic, octanoic, and decanoic carboxylic acids) from the longest esters they assayed, which corresponded to ethyl hexanoate, ethyl octanoate, and ethyl decanoate. However, these acids were not observed when the esters were incubated with saliva without the enzymatic activity. Despite this, the saliva EA was not measured in any of these works (3, 7).

In the case of wine, the effect of some matrix components, such as the phenolic compounds or ethanol in the oral release of wine esters is reported (21, 22), which could also be related to the oral metabolism by esterases. Regarding phenolic compounds, a recent study showed an increase of the ethyl hexanoate released into the headspace (above 60% more), when the wine-saliva systems were incubated in presence of phenolic compounds. This might suggest the inhibition of salivary esterase enzymes by polyphenols, thus, minimizing ester hydrolysis and as consequence, increasing the headspace concentration of this odorant molecule (23). In this way, the inhibition of natural flavonoids on the human carboxylesterases and carbonic anhydrases is widely investigated (24–29). However, these findings are yet to be proven for saliva esterase. Regarding ethanol, different effects in the oral ester release are observed depending on the ethanol content of wine (from 0.5 to 10%), the length of the carboxylic ester and the analytical approach to monitor oral aroma release, and the inter-individual variation (22). For instance, in the immediate oral release, an increase in the ethanol content showed an increase (up to 20%) in the release of small esters (ethyl butanoate and ethyl pentanoate), but a decrease (up to 20%) in the release of large esters (ethyl octanoate and ethyl decanoate), while in the prolonged oral release, ethanol showed an increase (up to 150%) in the release of all the carboxylic esters assayed (22). However, the impact of ethanol on saliva EA remains unexplored.

For all the above, this study aimed to provide insight into the potential role of saliva EA during wine oral processing. To do so, the specificity of saliva esterases toward six different types of carboxylic esters with different chain lengths and representative of the wine volatile profile was investigated in the SS and NSS samples from 10 individuals. Additionally, the inhibitory effect of single wine phenolic compounds (catechin, quercetin, kaempferol, myricetin, and resveratrol), and mixtures [grape seed extract (GSE), the monomers and oligomers purified fraction of this GSE (GSEM and GSEO), and a red wine extract (RWE)] in saliva EA, was assessed. Finally, the inhibitory effect of wine in the saliva EA was evaluated in more similar conditions to those found in the mouth during wine consumption that means under acidic pH and in the presence of ethanol (11.3%).

## Materials and Methods

### Saliva Collection

**Figure 1:**
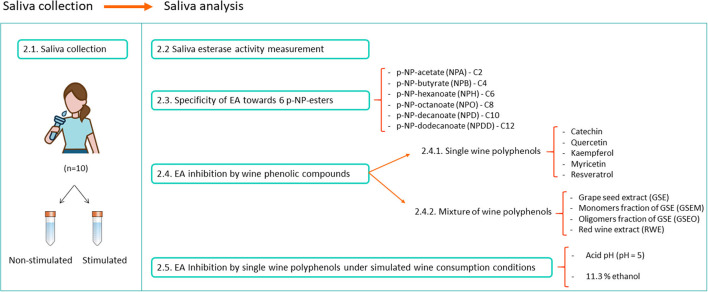
The experimental procedure followed for the saliva collection and saliva esterase activity (EA) assays.

The NSS and SS from the 10 volunteers (five women and five men) between 25 and 67 years of age were collected in the morning between 9 and 11 a.m. and for 5 min (Figure 1). The NSS was naturally collected in 15 ml centrifuge tubes (VWR, PA, USA), while for the SS, the volunteers chewed a piece of Parafilm™ during the collection (30). After this, the saliva samples were centrifuged at 15,000 g and 4°C during 15 min (31). The supernatants from the NSS and SS were pooled into two different tubes. The pH was determined in both the saliva mixtures SS (pH = 7.7) and NSS (pH = 7.6). Then, aliquots of 1.5 ml of saliva were prepared in Eppendorf tubes (VWR, PA, USA) and were frozen (−80°C) until the experiments. This storage technique has been previously proven not to affect the saliva EA (32). Figure 1 shows a schematic summary of the experimental procedure.

The volunteers did not report oral diseases. The donors were not allowed to eat or drink anything 1 h before the saliva collection. The participants were informed about the purpose of the study, and they gave their written consent to participate. These experiments were authorized by the Bioethical Committee of the Spanish National Council of Research (CSIC), Spain.

### Saliva EA Measurement

The saliva EA was measured following the colorimetric method previously described in the study of Pérez-Jiménez et al. (32) by using six different p-nitrophenyl (p-NP)-linked esters substrates provided by Sigma-Aldrich (Germany) and TCI Europe (Belgium), as detailed in the following section. The reaction mixture was composed of 25 μl of Tris 50 mM (pH = 8), 74 μl of saliva, and 25 μl of McIlvaine buffer (pH = 5) with the corresponding p-nitrophenyl (p-NP) ester substrate at 1.4 mM. The buffers Tris 50 mM (pH = 8)and McIlvaine (pH = 5) were prepared according to the procedure described by Pérez-Jiménez et al. (32). Tris was provided by GE Healthcare Uppsala, Sweden, and the reagents for McIlvaine buffer were provided by Panreac Química S.A., Spain (citric acid), and Merck. Darmstadt, Germany (Na_2_HPO_4_·12H_2_O). The pH of the reaction mixture was 6.7.

The EA determinations were performed in m96 microplates (VWR, PA, USA). To do so, the microplates were incubated for 35 min at 36°C, and the absorbance (λ = 410 nm) was measured every 5 min (eight times in total) by using a microplate spectrophotometer (Multiskan™ FC Microplate Photometer, Thermo Fischer Scientific, MA, USA). The EA was calculated as the units of EA (UI) per min (14). One unit of EA was determined as the absorbance change of 0.01 optical density. The saliva EA was measured three times.

### Specificity of Saliva EA

To determine the specificity of the saliva EA toward different carboxylic esters, six p-NP-linked esters were employed as substrates: p-NP-acetate (NPA), p-NP-butyrate (NPB), p-NP-hexanoate (NPH), p-NP-octanoate (NPO), p-NP-decanoate (NPD), and p-NP-dodecanoate (NPDD) (Figure 1). They differed among their hydrocarbon chain length (from C2 to C12, respectively). Six different 70 mM stock solutions (one per p-NP-ester) were prepared in 5 ml of methanol (Lab-Scan, Poland). Then, 1 ml of these 70 mM stocks were made up to 10 ml with McIlvaine buffer pH = 5, to obtain a 1.4 mM final concentration of the p-NP-esters in the microplate well.

### EA Inhibition by Wine Polyphenols

#### Single Wine Polyphenols

The inhibitory effect of five single wine polyphenols, such as catechin (Sigma, MO, USA), quercetin (Sigma), kaempferol (Extrasynthèse, France), myricetin (Extrasynthèse), and resveratrol (Extrasynthèse), was assayed (Figure 1). These wine phenolic compounds were selected for their inhibitory effect exerted on the human esterases from different tissues (liver and microsomes) other than saliva (24, 25, 27, 28, 33). Individual polyphenols were tested at the maximum concentration they can be present in wines as reported in previous studies (Table 1). For this, five different solutions (one per single polyphenol) were performed in Tris 50 mM pH = 8. As a positive control for the esterase inhibition, bis(4-nitrophenyl) phosphate (B4NP) (37) (TCI Europe, Belgium) was added at a final concentration of 0.1 mM into the well, which was selected in the previous trials. For this, a solution of 0.17 mg/ml of B4NP was prepared in Tris 50 mM (pH = 8).

**Table 1:** Concentration of the single flavonoids solutions in Tris 50 mM pH = 8 assayed in this experiment and maximum values of concentration of single wine flavonoids described in the literature.

**Flavonoid**	**Concentration (mM) in the microplate well**	**Concentration (μg/mL) in the microplate well**	**pH of Tris solutions**	**Max. concentration found in wine (mg/mL)**
Catechin	1.34	389	8.44	0.39^a^
Quercetin	0.11	31.7	8.41	0.0316^b^
Kaempferol	0.013	3.7	8.24	0.0036^b^
Myricetin	0.056	17.8	8.21	0.0179^a^
Resveratrol	0.12	27.4	8.21	0.0278^c^

a *Frankel et al. (34)*,

b *Rossouw and Marais (35)*,

c *Vitrac et al. (36)*.

#### Mixtures of Wine Polyphenols

In a further assay, the effect of four mixtures of wine polyphenols was evaluated in the saliva EA (Figure 1). For this, four different commercial phenolic extracts were employed: a GSE (Vitaflavan^®^), GSEM and GSEO, and an RWE) (Provinols™). The GSE and their purified fractions (GSEM and GSEO) were from Les Dèrives Resiniques & Terpéniques, S.A. (France), while RWE was from Safic-Alcan Especialidades, S.A.U. (Barcelona). The individual phenolic composition for GSE is shown in Sánchez-Patán et al. (38) for GSEO and GSEM in Cueva et al. (39), and for RWE, the composition is shown in Sánchez-Patán et al. (40). To evaluate the effect of the four mixtures of wine polyphenols on the saliva EA, different amounts of the four extracts (GSE, GSEO, GSEM, and RWE) were dissolved in Tris (50 mM, pH = 8). The selected concentrations of the four extracts in the well were selected to obtain the maximum concentration of every single phenolic compound (catechin, quercetin, kaempferol, mirycetin, and resveratrol) that has been described in wines (Table 1). The concentration and pH of the phenolic extract solutions in Tris are shown in Table 2.

**Table 2:** Concentration, pH, and composition of single wine flavonoids included in the polyphenolic extract solutions prepared in Tris 50 mM, pH = 8.

**Name**	**pH**	**mg of phenolic extracts (in 25 mL of Tris)**	**Catechin (μg)**	**Quercetin (μg)**	**Kaempferol (μg)**	**Myricetin (μg)**	**Resveratrol (μg)**
GSE	8.08	0.7	52.22	–	–	–	–
GSEM	8.06	0.4	54.19	–	–	–	–
GSEO1	8.00	1.8	46.08	–	–	–	0.77
GSEO2	8.09	8.0	204.80	–	–	–	3.42
RWE1	8.04	0.2	1.98	4.48	0.01	0.51	–
RWE2	8.09	12.25	121.28	274.40	0.45	31.24	–
RWE3	8.00	0.8	7.92	17.92	0.03	2.04	–
RWE4	8.04	4.8	47.52	107.52	0.18	12.24	–

### Inhibition of EA by Wine Phenolic Compounds Under the Simulated Wine Consumption Conditions

To check the inhibitory effect of wine flavonoids toward saliva EA in conditions more similar to those of wine consumption, a new experiment was carried out. For this, NPB (four carbons) and catechin were selected among the six ester substrates and the five single wine polyphenols (Figure 1). The inhibitory effect of catechin was evaluated in the presence of ethanol [11.3% (v/v)] and under more acidic pH conditions (pH = 5). These assays were carried out in both the SS and NSS samples.

To evaluate the effect of ethanol, 14 μl of absolute ethanol (Panreac Química S.A., Barcelona, Spain) was added to the microplate well. To maintain the final volume of the reaction mixture (124 μl), the volumes of the reagents were recalculated: 21.3 μl Tris (50 mM, pH = 8), 67.4 μl saliva, 21.3 μl McIlvaine buffer (pH = 5), and 14 μl ethanol.

To evaluate the wine tasting conditions in the terms of acidic pH on EA, a preliminary assay was performed to know the salivary pH after the wine tasting. The results are shown in Supplementary Figure 1. It was found that the pH of saliva immediately after wine rinses decreased to 5, thus, pH = 5 was selected to simulate the wine consumption conditions.

Then, the pH of the reaction mixture in the well was modified by replacing the Tris (50 mM, pH = 8) buffer with the McIlvaine (pH = 4.56) buffer. The effect of changing the buffer was previously checked, showing that the EA measurements were not affected.

To evaluate the effect of both factors (ethanol 11.3% and pH = 5), the volumes of the reaction mixture were recalculated as explained before and the Tris (50 mM, pH = 8) buffer with the McIlvaine (pH = 4.56) buffer. Then, the EA was measured following the same procedure as explained in section Saliva esterase activity measurement.

### Statistical Analysis

One-way ANOVAs and Tukey's tests for mean comparison were applied to check for the significant differences (*p* < 0.05) in the EA: between the SS and NSS, to check the inhibitory effect of the single and mixtures of wine polyphenols and to determine the influence of ethanol (11.3%) and acidic pH (pH = 5) in the inhibitory effect of catechin. To do so, version 2020.3.1 of the XLSTAT (Addinsoft, Paris, France) software was used.

## Results and Discussion

### Specificity of Esterase Enzymes by Carboxylic Esters in the SS and NSS

**Figure 2:**
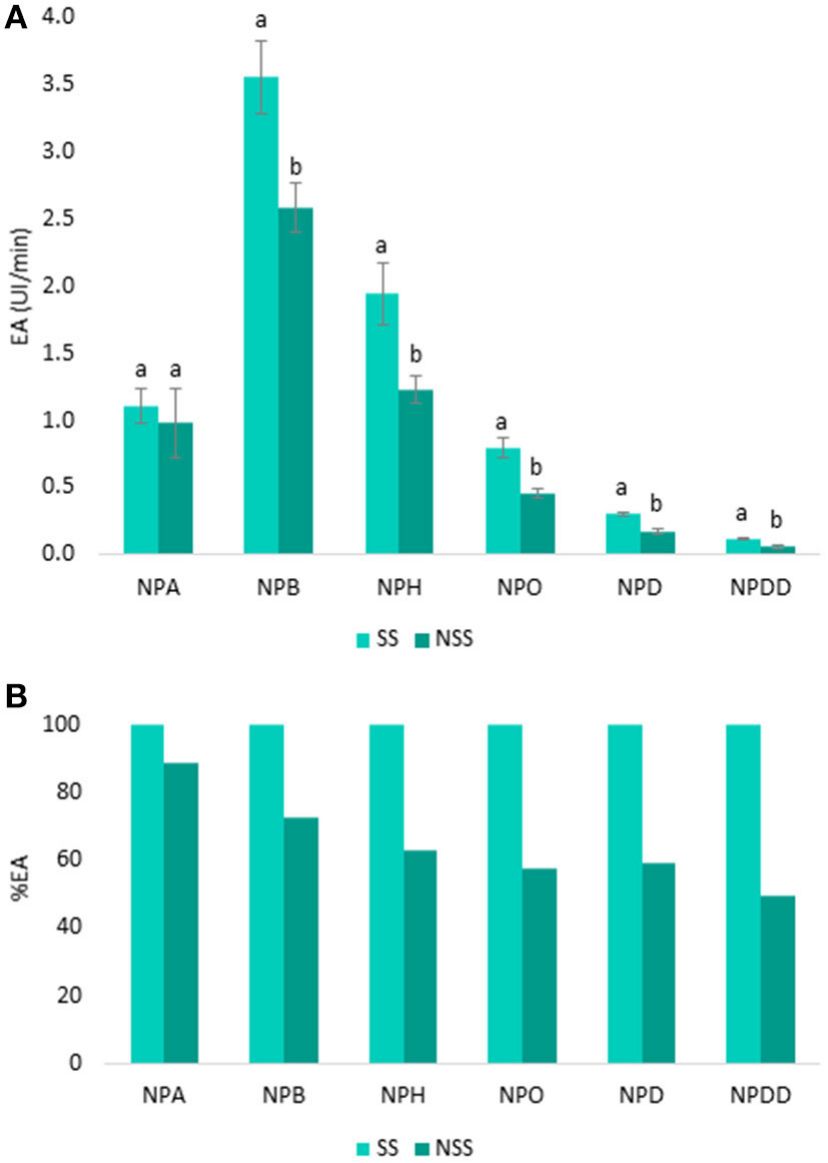
Specificity of the esterase activity in UI/min in stimulated saliva (SS) and non-stimulated saliva (NSS) toward six p-NP-esters: p-NP-acetate (NPA), p-NP-butyrate (NPB), p-NP-hexanoate (NPH), p-NP-octanoate (NPO), p-NP-decanoate (NPD), and p-NP-dodecanoate (NPDD) **(A)**. Letters above the bars denote significant differences (*p* < 0.05) between SS and NSS from Tukey's test. In **(B)**, the EA from SS was considered as 100%, the EA percentage from NSS was then calculated.

To determine the specificity of esterases from both SS and NSS for carboxylic esters, the EA was measured by using six p-NP-ester substrates (NPA, NPB, NPH, NPO, NPD, and NPDD) that differed in carbon chain length (from C2 to C12). The mean values of EA (UI/min) and SD are shown in Figure 2.

As it can be seen (Figure 2A), the EA was higher in the SS than in the NSS for all the p-NP-esters, and these differences in the EA between the two saliva types increased from 11 to 50% as the length of the ester chain increased. In Figure 2B, the EA from SS was considered as 100%, and from here, the percentage of EA from NSS was calculated. As it can be seen, the EA from SS measured with smaller esters (NPA, NPB, and NPH) was between 11 and 37% higher than EA from NSS with the same esters (Figure 2B). In the case of longer esters (NPO, NPD, and NPDD) the differences in the EA between SS and NS were even higher (42–50% higher in SS than in NSS). The main difference between the two saliva types is that SS is mostly produced by the parotid gland, while NSS is produced by sub-mandibular glands (41). Therefore, these results indicated that the saliva EA mainly proceeds from parotid saliva, which is in agreement with the previous findings (42). Apart from parotid saliva, epithelial cells and oral microorganisms are proposed as the other important sources of saliva esterase (43, 44).

On the other hand, Figure 2A shows that the higher the carbon chain length of the ester, the lower the EA, independent of the type of saliva (SS and NSS). This trend was observed for all the p-NP-ester substrates, except for the shortest ester NPA (two carbons), in which the EA from both the saliva types was between 60 and 70% lower than in the NPB (four carbons). Greater hydrolysis toward butyrate than acetate esters by carboxylesterases from saliva (9, 15) and liver (45) is seen before. Apart from NPA, as the size of the ester increased, the EA decreased. Therefore, the NPB substrate showed the highest EA in the two saliva types, which was 50–55% greater than the activity using NPH (six carbons), and up to 97% higher than in the case of longer chain esters, such as NPD and NPDD (10 carbons or more). A lower EA toward the longer esters instead of smaller esters is shown by other authors in the case of human liver carboxylesterases (46) and carbonic anhydrases (47, 48). In the case of human liver carboxylesterases, an increase in the affinity constant between the ester and the enzyme as the hydrophobicity (log P) of the ester increased is shown (45, 46). A reduction in the maximum rate of reaction (Vmax) is produced as the size of the product (carboxylic acid) increased, since more hydrophobic products are located in the active site gorge, reducing the activity of the enzyme (45, 46). Therefore, on one hand, it seems that there is a greater affinity of esterases for the more hydrophobic esters, which could lead to the larger carboxylic acid products, which in turn, decreases the reaction rate, and thus, the activity of the enzyme. But, on the other hand, the activity of esterases increases for the smaller esters, which, although have a lower affinity for the enzyme, lead to small carboxylic acid hydrolysis products, which do not affect the activity rate of the esterase enzymes, contrarily to what happens with the long esters. Thus, the size of the degradation products seems more important for the enzymatic reaction rate than the affinity between the enzyme and the ester. Therefore, for small esters (such as NPA and NPB), the higher the log P of the ester, the higher the EA, while as the size of the carboxylic acid product increases (six carbons or more), the EA decreases. Although this premise was not previously proven for saliva esterases, it could be a plausible hypothesis to explain these results. Additionally, this hypothesis might explain the higher persistence of long than short chain esters in the breath after wine consumption (49).

### Inhibition of EA by the Wine Phenolic Compounds

Prior to the inhibitory assay of EA by the wine phenolic compounds, the use of B4NP as a positive control for the inhibition of saliva EA was checked, as B4NP is widely known to be a specific inhibitor of carboxylesterases (37, 50, 51). For this, B4NP was added to SS and NSS at different concentrations in the microplate wells (from 0.05 to 0.8 mM), and the EA was then measured following the same procedure as previously described.

The results showed that the inhibitory effect of B4NP was not dose-dependent as seen previously (37, 51, 52). The maximum inhibitory effect of B4NP toward EA was found at 0.05, 0.1, and 0.2 mM concentrations of B4NP (Supplementary Figure 2). At these concentrations, B4NP inhibited ~65–70% of EA in both the saliva types. These results confirmed that, although there are more enzymes involved in salivary EA (15, 18), carboxylesterases are the main enzymes that contribute to this activity in saliva (9, 17).

Therefore, 0.1 mM concentration of B4NP was selected for the following assays, which is within the range that was previously used by other authors (37, 50, 53, 54). This concentration provided the highest EA inhibition (65 and 69% in SS and NSS, respectively).

#### Inhibition of Saliva EA by the Single Wine Polyphenols

**Figure 3:**
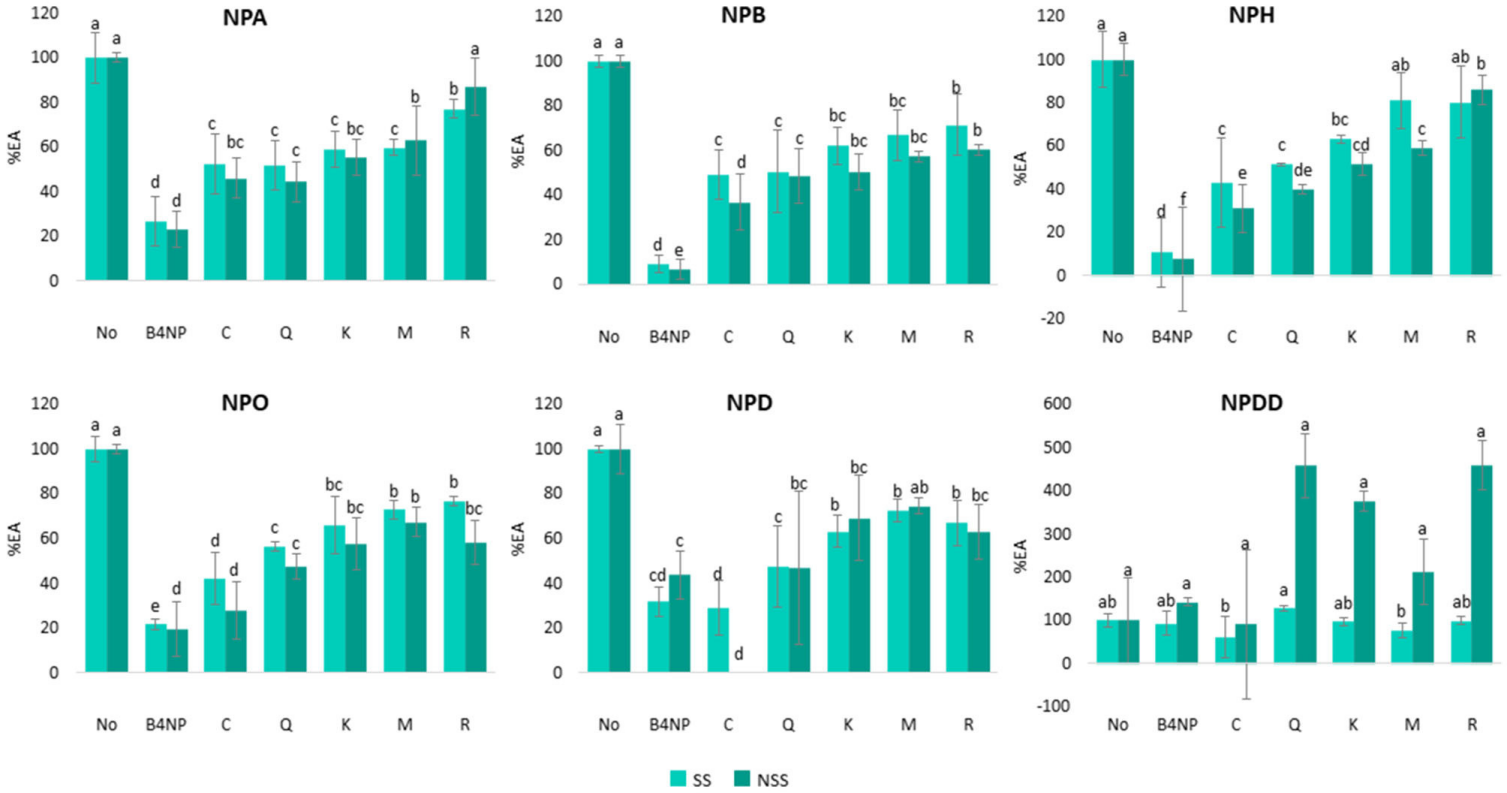
Percentage of EA (%EA) in SS and NSS toward six p-NP-esters with different carbon chain length (from C2 to C12) in the presence of five single wine polyphenols: catechin (C), quercetin (Q), kaempferol (K), mirycetin (M), and resveratrol (R) assayed at the maximum concentration found in the wines. B4NP 0.1 Mm was employed as a positive control. Different letters above the bars mean significant differences (*p* < 0.05) from Tukey's test.

The EA inhibitory effect of single wine polyphenols (Table 1) was previously reported to inhibit different types of human esterases (24, 25, 27, 28, 33) was checked. The results are shown in Figure 3.

As it can be observed (Figure 3), all the single phenolic compounds exerted an inhibitory effect from 13 to 72% toward EA in both the SS and NSS and in the presence of all the p-NP-ester substrates, except in the case of the substrate NPDD (12 carbons). For NPDD in the SS, only the presence of catechin and myricetin exerted a significant (*p* < 0.05) inhibitory effect in EA compared with the control saliva (without phenolic compounds). Nonetheless, for the same substrate, NPDD, but in the NSS, there were no significant differences in the EA among the saliva samples with or without the phenolic compounds. The odd results obtained for NPDD could be due to the low EA proven for this substrate (values lower than 0.1 U/min) (Figure 2), which could explain the large variations observed in Figure 2. Due to this reason, the results from NPDD are not considered.

Apart from this, the inhibition of EA by the five single wine phenolic compounds at the concentration assayed, which were within the typical wine values, was statistically significant (*p* < 0.05) in all the cases, except for resveratrol in the NSS. For this phenolic compound, the EA toward NPA (two carbons) was also reduced (13%) compared with the control saliva, but this reduction was not statistically significant (Figure 3). Interestingly, the inhibitory effect on saliva EA was different depending on the concentration and the type of phenolic compound (Table 1, Supplementary Figure 3). Similarly, the addition of the flavan-3-ol catechin, showed in general, the highest EA inhibition (48–100%) in both SS and NSS, and considering all the ester substrates, which was the single polyphenol added at the highest concentration (389 μg/ml). The flavonols (quercetin, kaempferol, and myricetin), showed an intermedium esterase inhibitory effect (20–60%) in both the saliva types. These compounds were added at much lower concentrations: 31.7 μg/ml (quercetin), 3.7 μg/ml (kaempferol), and 17.8 μg/ml (myricetin), as they generally occur in wine. Finally, the addition of 27.4 μg/ml of resveratrol, which is a stilbene, produced the lowest EA inhibition (13–43%), in the two saliva types. These results might be related to both the concentrations assayed and the structural differences of these phenolic compounds. The catechin was added at the highest concentration, while quercetin, myricetin, and resveratrol were added at more similar concentrations (between 17 and 32 μg/ml) and kaempferol was added at the lowest concentration (Table 1). Regarding the structure of the single wine polyphenols, the main difference between the flavonoids and stilbenes is the number of molecular rings, which are three and two, respectively. While between the flavan-3-ol and flavonols, the main difference was the type of C ring (Supplementary Figure 3). Within the flavonol group, they differ in the hydroxy groups in C-3′ and C-5′ positions (Supplementary Figure 3). Therefore, these results showed that the inhibition exerted by the wine polyphenols was related to both their concentration and molecular structure.

The inhibitory effect of catechin (24, 27), quercetin (24, 25, 28, 55), kaempferol (28, 55), and resveratrol (27) on the human carboxyl esterases and carbonic anhydrases is previously shown. In addition, the inhibitory effect of myricetin is reported toward acetylcholine esterase (33). Although the inhibitory mechanism of the five phenolic compounds employed in this work toward saliva esterases is not investigated so far, the previous works using other natural flavonoids (e.g., 5,6-dihydroxyflavone and luteolin) have studied the inhibition of human carboxylesterases using the molecular docking approaches (28, 56). The inhibition was explained by the interactions between the amino acid residues of the protein-ligand complex with specific chemical groups (such as hydroxy or ketone carbonyl groups) at specific carbon positions (e.g., C-4′, C7) of the flavonoid molecule. This leads to the formation of hydrogen bonds between the flavonoids and the active site of the enzyme, thus reducing the binding with its substrate (ester molecule) (56). Although in the above-mentioned works, the esterases were from human tissues, such as liver or microsomes (28, 56), which are very different from the esterases from saliva, this explanation could also be valid in explaining the inhibition exerted by the assayed phenolic compounds in the saliva esterases.

The inhibitory effect of each type of wine phenolic compound varied depending on the type of saliva (SS and NSS) and the type of p-NP-ester substrate (NPA, NPB, NPH, NPO, NPD, and NPDD) (Figure 3). Regarding the type of saliva, a significantly (*p* < 0.05) higher inhibitory effect (from 2 to 28%) in NSS compared to SS was observed. Despite this, the differences in the inhibition between SS and NSS were in general lower than 15%, although in some of the cases (myricetin in NPH, reseveratrol in NPO, and catechin in NPD), these differences increased up to 28%.

As stated before, the effect of the wine phenolic compounds varied depending on the p-NP-ester substrate. In the case of catechin, its inhibitory effect toward saliva EA increased as the length of the ester increased (from 2 to 10 carbons). The inhibition exerted by catechin was 24% (in SS) and 46% (in NSS) higher in NPD (10 carbons) compared with NPA (two carbons) (Table 3). On the contrary, in the case of quercetin and kaempferol, their inhibitory effect toward saliva EA was similar for all the ester substrates (variations lower than 7 and 9% toward the six p-NP esters). In the case of myricetin and resveratrol, their inhibitory effect also varied among the ester substrates (between 10 and 29%) in the two saliva types, although these differences did not seem to be related to the chain length of the ester.

**Table 3:** Inhibition (%) of EA by single wine polyphenols at the maximum concentrations typically found in wines, toward five p-NP-esters (NPA, NPB, NPH, NPO, and NPD) with different carbon chain length (from C2 to C10) determined in stimulated and non stimulated saliva (SS and NSS).

	**NPA**	**NPB**	**NPH**	**NPO**	**NPD**	**NPA**	**NPB**	**NPH**	**NPO**	**NPD**

	**Stimulated saliva (SS)**					**Non stimulated saliva (NSS)**				
Catechin	47.60	50.69	56.82	57.87	71.13	53.87	63.00	68.85	72.28	100.00
Quercetin	48.25	49.30	48.47	43.69	52.65	55.54	51.47	59.77	52.67	53.26
Kaempferol	41.08	37.73	36.66	34.12	36.73	44.42	49.34	48.43	42.43	30.82
Mirycetin	40.14	33.10	18.77	27.11	27.62	37.02	42.63	40.81	32.67	25.44
Resveratrol	22.80	28.36	19.66	23.36	33.05	13.05	39.65	13.72	41.74	37.05

#### Mixture of Wine Polyphenols

**Figure 4:**
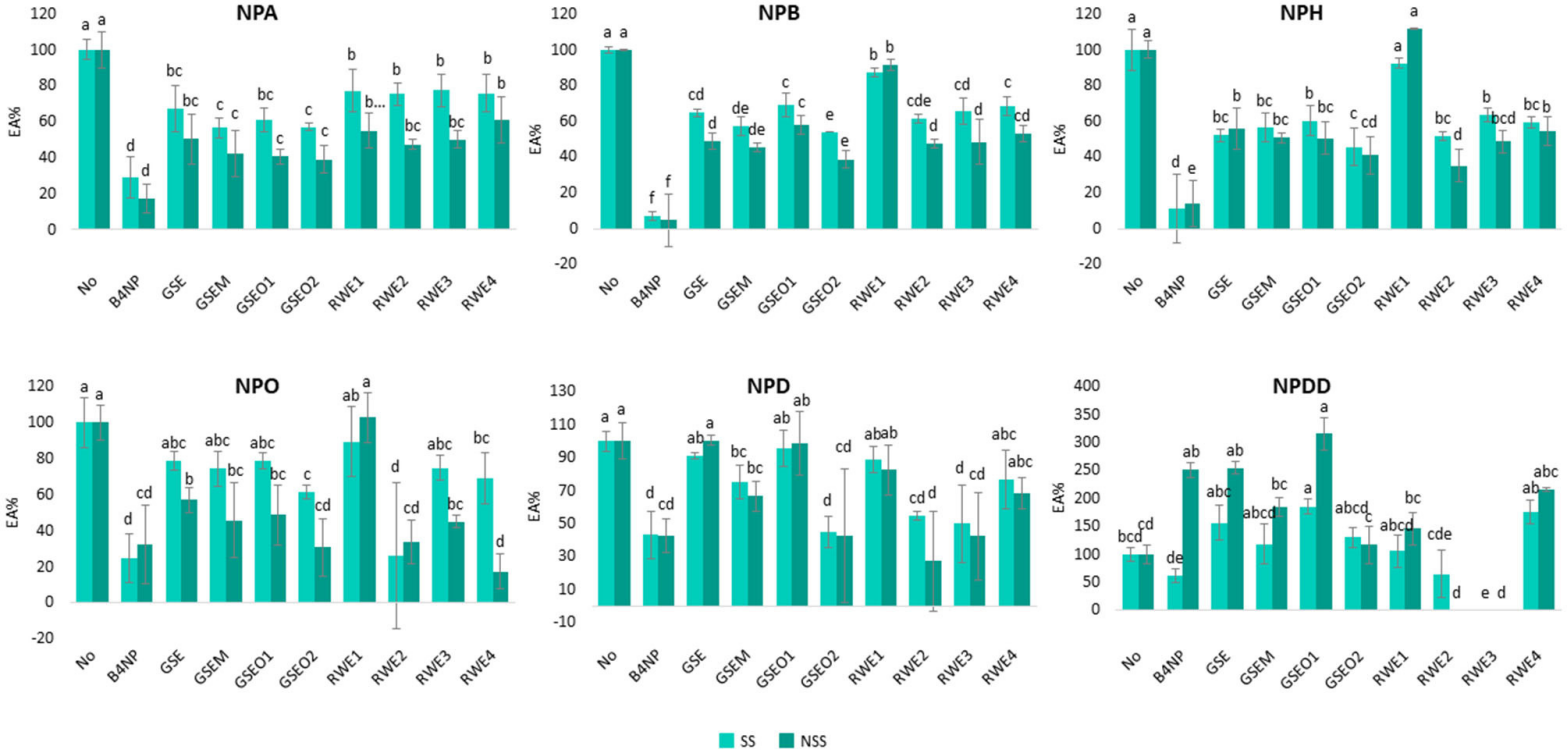
Percentage of EA determined in SS and NSS toward six p-NP-esters with different carbon chain lengths (from C2 to C12) in the presence of four phenolic extracts (GSE, GSEM, GSEO, and RWE) added at different concentrations to the microplate well-depending on the maximum concentration of each of their phenolic components found in the red wines (Table 2). Different letters above the bars mean significant differences (*p* < 0.05) from Tukey's test.

In a further step, the inhibitory effect of four mixtures of wine polyphenols (phenolic extracts) on the saliva EA was evaluated. For this, different phenolic extracts were employed (GSE, GSEM, GSEO, and RWE) at different concentrations, which were selected considering the maximal concentration of the main individual phenolic components that naturally occur in the wines (Table 2). The percentages of EA in the SS and NSS in the presence of the mixtures of the wine polyphenols are depicted in Figure 4.

As shown in Figure 4, all the mixtures of wine polyphenols exerted an inhibitory effect on the saliva EA in both SS and NSS, and this inhibition was statistically significant (*p* < 0.05) for most of the samples. Apart from this, and similar to the results found in the section earlier for the single phenolic compounds, a large variation in the percentage EA using NPDD (12 carbons) was noticed. Because of this and as previously explained, the results from NPDD were excluded from the following discussion.

However, the inhibitory effect of the mixture of wine polyphenols also varied depending on the type of saliva (SS and NSS), the type of p-NP-ester (NPA, NPB, NPH, NPO, NPD, and NPDD), and the type of phenolic extract (GSE, GSEM, GSEO, and RWE) (Figure 4).

As previously observed for the single wine polyphenols, a significantly (*p* < 0.05) higher inhibitory effect of the polyphenol extracts was found in NSS compared with the SS. The differences in the inhibition between SS and NSS were, in general, lower than 28%, although in the case of the addition of RWE at the highest concentration assayed (RWE4) in NPO, these differences increased up to 51%.

Regarding the type of extract, in the SS, the GSEO2 extract showed the highest inhibitory effect (inhibition >43%), except in NPO, which was the RWE2 extract (74% inhibition), and in NPA, which was GSEM (43% inhibition). Similarly, in the NSS, the GSEO2 and RWE2 extracts showed the highest EA inhibition (inhibition higher than 60%), except in the case of NPO in which RWE4 showed the highest EA inhibition (82% inhibition). Interestingly, the extracts which showed the highest EA inhibition in both the types of saliva (GSEO2, RWE2, GSEM, and RWE4), contained more catechin in their composition (Table 2). Among them, GSEO2 and RWE2, which showed in general, the highest EA inhibition, were those with the highest concentration of catechin, 204.8 and 124.28 μg/ml in the case of GSEO2 and RWE2, respectively. On the other hand, the RWE1 extract showed in general the lowest EA inhibition (inhibition lower than 23%) toward all the p-NP-esters, except in NSS. In NSS, the lowest EA toward NPA was exerted by RWE4 (39% inhibition). Interestingly, the RWE1 extract showed the lowest catechin concentration (1.98 μg/ml). Therefore, the concentration of catechin in the extracts was a key aspect in explaining the inhibition of salivary EA. Therefore, the higher the concentration of catechin, the higher the EA inhibition.

Regarding the effect of the phenolic extract concentration, differences were observed in the EA inhibition. For example, in the case of GSEO extracts (GSEO1 and GSEO2), a greater EA inhibition (up to 55%) was observed when the extract was assayed at higher (GSEO2) than lower (GSEO1) concentrations in both the saliva types (SS and NSS) and toward the six p-NP-ester substrates. Since GSEO is composed of 70% flavan-3-ols, these results suggest that as the concentration of flavan-3-ol increased, the EA decreased. Contrarily, in the case of RWE, which was added at four different concentrations (RWE1, RWE2, RWE3, and RWE4), no relationship between the concentration of the extract and the inhibition of EA was observed. This indicated that in the case of anthocyanins (significant phenolic compounds in RWE), the inhibitory effect toward saliva esterases was not concentration-dependent.

Therefore, these results showed that the mixture of wine polyphenols exerted an inhibitory effect on the saliva EA, which varied depending on the concentration and on the phenolic composition of the extract.

### EA Under the Wine Consumption Conditions

Finally, to evaluate how EA could be modified during the wine tasting conditions and if phenolic compounds could also inhibit this enzymatic activity, a new assay was set up. For this, the EA was first measured in the presence of ethanol and acidic conditions. The results (Supplementary Figure 1) showed, as expected, that salivary pH decreased (average values from 6.7 to 5.6,) immediately after wine expectoration (0 s). Interestingly, 30 s after wine rinsing, the salivary pH was practically the same (pH = 6.3) as the baseline pH (pH = 6.7), which indicated that a short recovery time is needed by saliva to return to its baseline pH value, as observed in the previous works (57).

**Figure 5:**
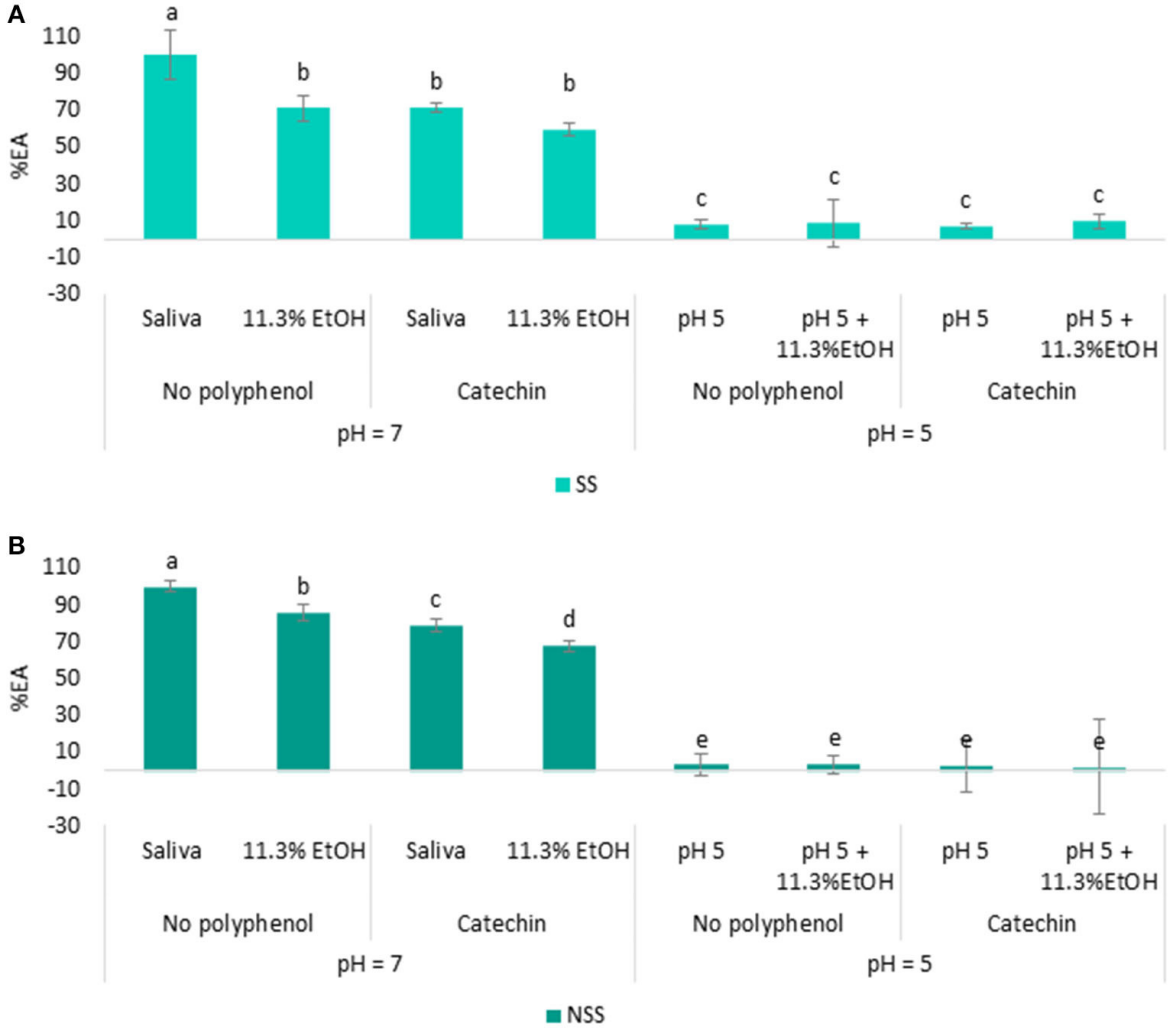
Percentage of EA determined in SS **(A)** and NSS **(B)** using wine consumption conditions: 11.3% ethanol, pH = 5 and with or without catechin.

The EA was then, measured in the saliva samples at acidic pH (pH = 5) or standard salivary pH (pH = 7), in the presence or absence of polyphenol (catechin), and also in the presence and absence of ethanol (11.3% v/v). The results, expressed as a percentage of EA, are shown in Figure 5.

As it can be observed (Figure 5), the presence of ethanol has a significant effect on reducing the saliva EA. A decrease of about 29% in SS (Figure 5A) and 15% in NSS (Figure 5B) compared with control saliva (without ethanol) was observed. Although the presence of ethanol on saliva EA is scarcely investigated, the effect of acute ethanol consumption has been shown to significantly decrease the activity of other saliva enzymes, such as peroxidases and isozymes (58). The presence of ethanol or its metabolites could have inactivated the saliva esterases. The reduction in the EA from saliva could explain the higher persistence of carboxylic esters after the rinses with the wines supplemented with ethanol (5 and 10%) compared with the wines without ethanol (0.5%) observed in the previous works (22). In addition to this, the EA of the two saliva types also reduced about 12% for SS and 11% for NSS in the presence of both catechin and ethanol compared with the saliva without catechin and ethanol (Figure 5). These results showed that the decrease in saliva EA was higher when both the factors (catechin and ethanol) were evaluated at the same time than when they were evaluated individually.

Regarding salivary pH, the EA of both saliva types (SS and NSS) was significantly reduced by more than 90% when the pH of the well-decreased to 5 and in all the assayed conditions, with or without catechin and ethanol (Figure 5). These results indicated that under the acidic pH conditions (pH = 5), the ability of esterase enzymes to hydrolyze esters was compromised, which is in agreement with the previous findings, in which a pH between 7 and 8 has been established as the optimal pH for saliva esterases (16, 20, 59). Despite this, in a recent work, the authors determined the EA in saliva collected after mouth rinsing with an acidic (pH 3.5) water solution (32). These contradictory findings could be explained by the short time required for saliva to return to normal pH values, as previously observed in Supplementary Figure 1. Although the esterases barely showed enzymatic activity at acidic pH, saliva is constantly being generated and replenished, and as the pH of the oral environment return to the normal values within few seconds (Supplementary Figure 1), the esterases from saliva could also keep their activity. This suggests that the saliva EA might be reduced for a short time when saliva is in contact with the wine, but the EA activity can quickly be recovered when the pH of the saliva returns to more neutral values. This fact could be of great importance for the hydrolysis of esters remaining in the oral cavity during the wine tasting and thus, for the long-lasting wine aroma perception. Further studies focusing on the dynamic changes in the saliva composition and EA during a consumption episode will be achieved in future works to confirm this premise.

## Conclusions

This study showed for the first time the effect of wine polyphenols on the salivary EA from the SS and NSS and using six different ester substrates. The results showed a higher EA toward the smaller than the bigger esters. However, the addition of typical wine polyphenols individually or as a mixture, at natural concentrations found in the red wines decreased the saliva EA. This decrease was related to the concentration and the type of each phenolic compound. Among the single wine polyphenols, the flavan-3-ols (catechin) showed the highest EA inhibition followed by the flavonols (quercetin, kaempferol, and mirycetin) and stilbenes (resveratrol). The differences in the inhibitory effect of single polyphenols, depending on the concentration occurring in wine, but could also be related to their molecular structure (rings and hydroxyl groups). In the case of the mixtures of wine polyphenols, the highest EA inhibition was exerted by the oligomer GSE (GSEO2) and the RWE (RWE2), both assayed at the highest concentration, which were those that showed the highest concentration of catechin. While RWE at the lowest concentration assayed (RWE1), which presented the lowest catechin concentration, also exerted the lowest EA inhibition. In the simulated wine consumption conditions (11.3% ethanol and pH 5), the activity of saliva esterases was compromised, but the fact that saliva is constantly being generated and replenished, and, as the pH of the oral environment returns to the normal values within few seconds, salivary esterases could also keep their activity, and therefore play an important role in the long-lasting perception of esters during wine tasting. Overall, this study will contribute to better understand how aroma compounds might modify during the wine tasting, and how this could be related to the combined action of chemical wine composition and individual physiology, which could be explored as a strategy to get more personalized and appealing wine types.

## Data Availability Statement

The original contributions presented in the study are included in the article/Supplementary Material, further inquiries can be directed to the corresponding author/s.

## Ethics Statement

The studies involving human participants were reviewed and approved by the Bioethical Committee of The Spanish National Council of Research (CSIC). The participants provided their written informed consent to participate in this study.

## Author Contributions

MP-J: investigation, methodology, formal analysis, data curation, and writing—original draft. CM-G: data curation and writing—review and editing. MP-B: conceptualization, funding acquisition, supervision, and writing—review and editing. All authors have read and agreed to the final version of the manuscript.

## Funding

This research was funded by the Spanish MINECO and CAM through the PID2019-111734RB-100 and 2019T1/BIO13748 projects respectively. The funders had no role in the design of the study; in the collection, analyses, or interpretation of data; in the writing of the manuscript; or in the decision to publish the results.

## Conflict of Interest

The authors declare that the research was conducted in the absence of any commercial or financial relationships that could be construed as a potential conflict of interest.

## Publisher's Note

All claims expressed in this article are solely those of the authors and do not necessarily represent those of their affiliated organizations, or those of the publisher, the editors and the reviewers. Any product that may be evaluated in this article, or claim that may be made by its manufacturer, is not guaranteed or endorsed by the publisher.

## Supplementary Material

Supplementary Figure 1 Salivary pH measured before, immediately after wine rinsing (0 s), and each 30 s up to 120 s after wine rinsing. The average values and SD from three individuals are depicted.

Supplementary Figure 2 Inhibitory effect of B4NP at different concentrations in the microplate well (from 0.05 to 0.8 mM) against the esterase activity (EA) from stimulated saliva (SS) and non-stimulated saliva (NSS).

Supplementary Figure 3 Structure of the phenolic compounds employed in this study.

Supplementary Figure 4 Percentage of esterase activity (%EA) determined in saliva (SS and NSS), towards six p-NP-esters with different carbon chain length (from C2 to C12) in the presence of four phenolic extracts (GSE, GSEM, GSEO, and RWE). Concentration of each extract was selected considering the highest concentration of each individual phenolic constituents previously reported in wines (Table 2). Different letters above bars mean significant differences (*p* < 0.05) from Tukey test.

## Abbreviations

EA: esterase activity
p-NP: p-nitrophenyl
NPA: p-NP-acetate
NPB: p-NP-butyrate
NPH: p-NP-hexanoate
NPO: p-NP-octanoate
NPD: p-NP-decanoate
NPDD: p-NP-dodecanoate
SS: stimulated saliva
NSS: non-stimulated saliva
GSE: grape seed extract
GSEO: oligomers purified fraction of the grape seed extract
GSEM: monomers purified fraction of the grape seed extract
RWE: red wine extract.
