# Supplementary figures and images for: Specificity of Saliva Esterases by Wine Carboxylic Esters and Inhibition by Wine Phenolic Compounds Under Simulated Oral Conditions
Source: Front Nutr. 2021 Nov 4;8:761830. doi: 10.3389/fnut.2021.761830 (PMC8599952; doi:10.3389/fnut.2021.761830)

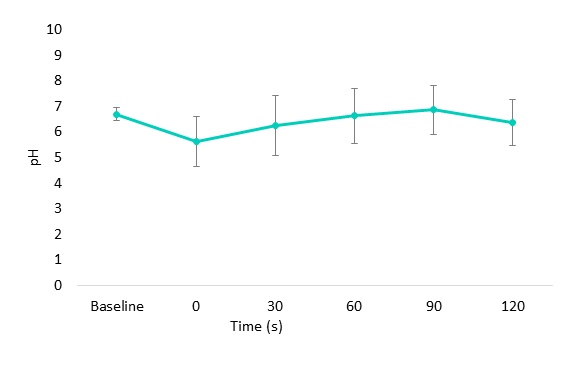

Supplement: Supplementary Figure 1 — Salivary pH measured before, immediately after wine rinsing (0 s), and each 30 s up to 120 s after wine rinsing. The average values and SD from three individuals are depicted. [file Image_1.TIFF]

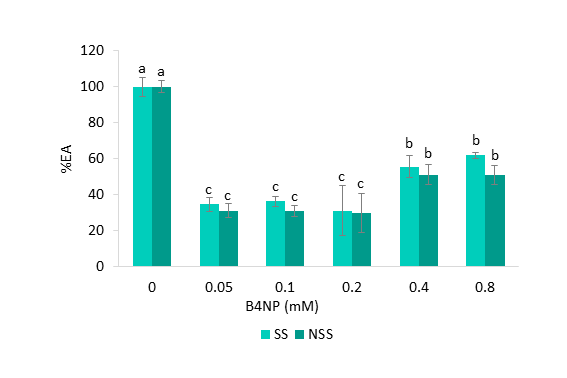

Supplement: Supplementary Figure 2 — Inhibitory effect of B4NP at different concentrations in the microplate well (from 0.05 to 0.8 mM) against the esterase activity (EA) from stimulated saliva (SS) and non-stimulated saliva (NSS). [file Image_2.TIFF]

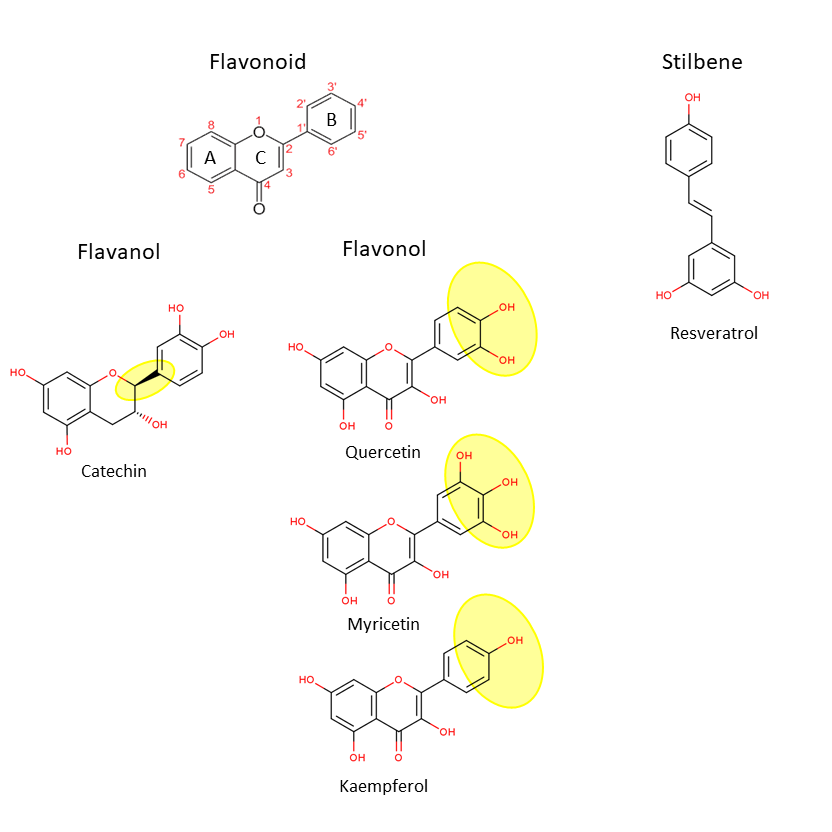

Supplement: Supplementary Figure 3 — Structure of the phenolic compounds employed in this study. [file Image_3.TIFF]

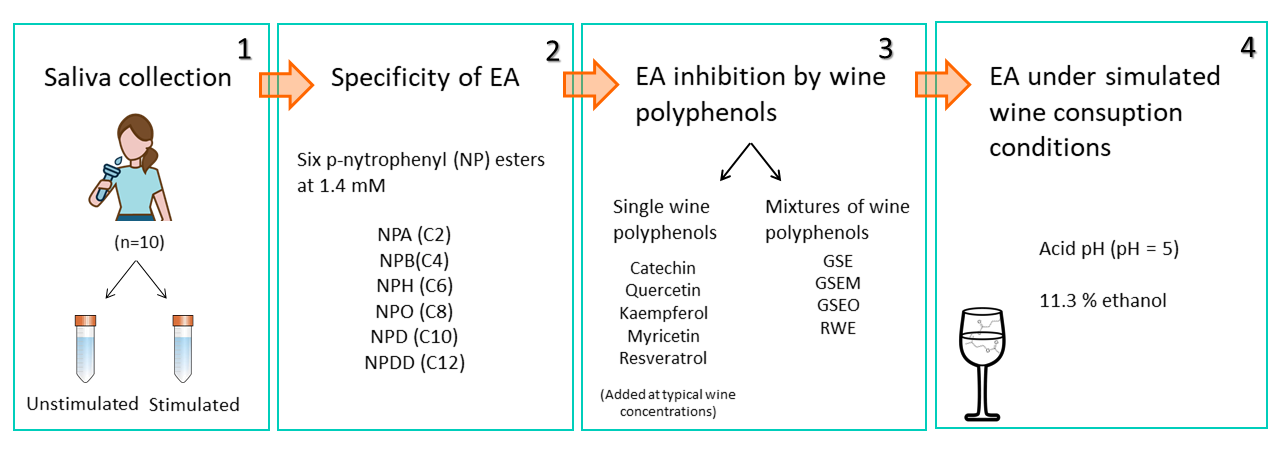

Supplement: Supplementary Figure 4 — Percentage of esterase activity (%EA) determined in saliva (SS and NSS), towards six p-NP-esters with different carbon chain length (from C2 to C12) in the presence of four phenolic extracts (GSE, GSEM, GSEO, and RWE). Concentration of each extract was selected considering the highest concentration of each individual phenolic constituents previously reported in wines (Table 2). Different letters above bars mean significant differences (p < 0.05) from Tukey test. [file Image_4.TIFF]
